# Editorial: The evolving picture of Ca^2+^ leak from endoplasmic reticulum in health and diseases

**DOI:** 10.3389/fphys.2023.1182455

**Published:** 2023-03-27

**Authors:** Adolfo Cavalié, Richard Zimmermann

**Affiliations:** ^1^ Experimental and Clinical Pharmacology and Toxicology, Pre-clinical Center for Molecular Signalling (PZMS), Saarland University, Homburg, Germany; ^2^ Competence Center for Molecular Medicine, Saarland University, Homburg, Germany

**Keywords:** calcium homeostasis, calcium leak, endoplasmic reticulum, Sec61 channel, translocon, tumor driver gene Sec62

## Introduction

**FIGURE 1:**
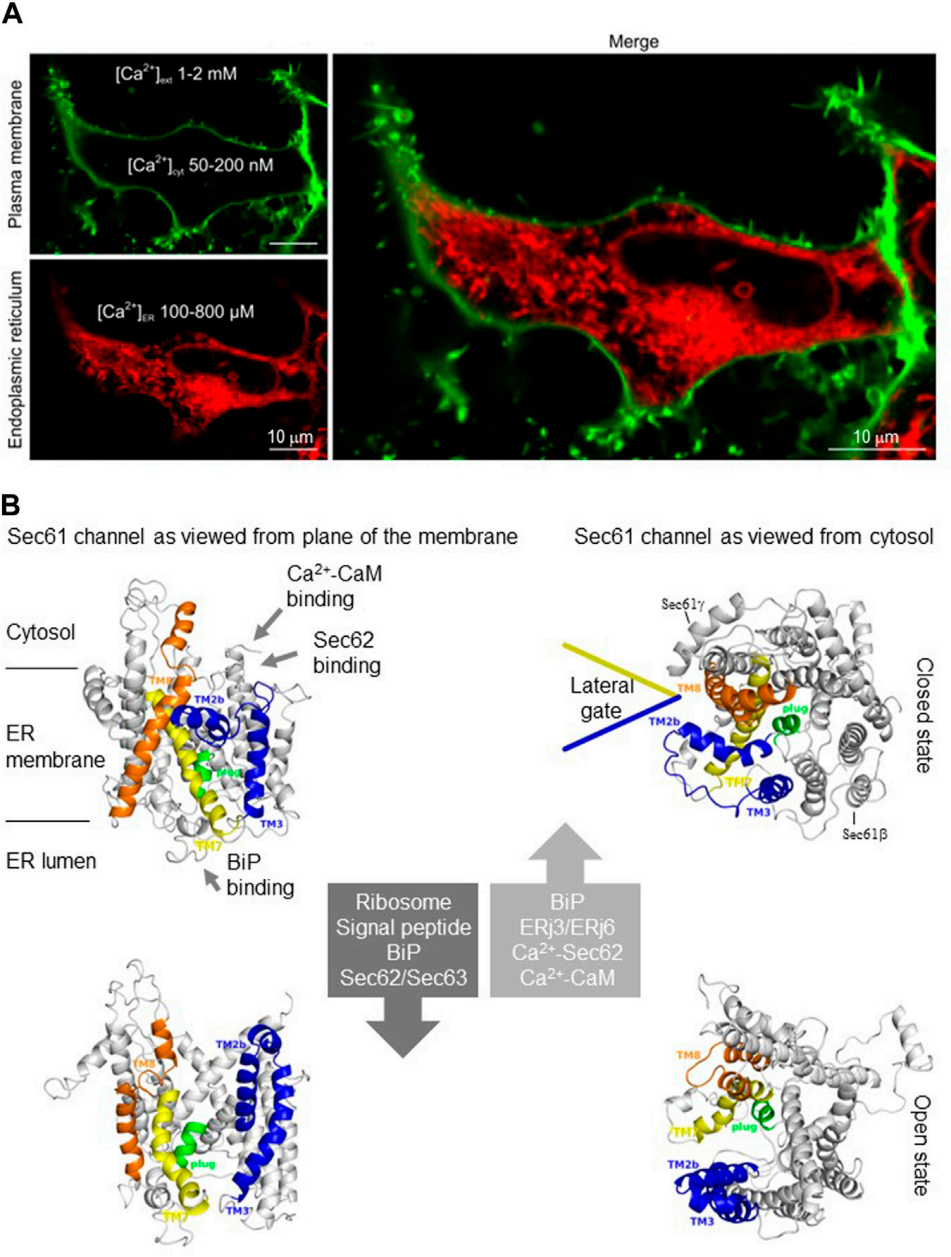
**I** The endoplasmic reticulum (ER) of nucleated human cells has major functions in cellular calcium homeostasis and contains the abundant and ubiquitous Sec61 channel. **(A)** The ER is shown here in a HEK293 cell after fluorescence microscopy after staining with ER-Tracker™ Red (BODIPY™ TR Glibenclamide), the plasma membrane was stained with CellMask™ Green Plasma Membrane Stain (details are given by Pick et al.). The image was kindly provided by Tillman Pick (Experimental and Clinical Pharmacology and Toxicology, Saarland University). **(B)** The Sec61 channel is shown in its modeled closed (top) and open (bottom) conformational states, as indicated (adopted from Lang et al., 2017). These two states are proposed to be in a dynamic equilibrium with each other. The fully open state of the Sec61 channel allows the initial entry of precursor polypeptides from the cytosol into the ER lumen and ER membrane, respectively. In addition, it allows the passive efflux of Ca^2+^ from the ER lumen into the cytosol after termination of the translocation process and, therefore, it can be quantified in live cell Ca^2+^ imaging in cytosol and ER lumen using ratiometric dyes and fluorescent proteins. Ca^2+^ efflux may also be possible in the transition state (not shown), which can be detected in the presence of Sec61 channel inhibitors such as Eeyarestatins or Mycolactone and may be identical to the so-called primed state that can be induced by ribosomes in co-translational- and by the Sec62/Sec63 complex in post-translational-transport (Gamayun et al., 2019; Bhadra et al., 2021).

The endoplasmic reticulum (ER) is one of the two main reservoirs for releasable Ca^2+^ in the cell and usually maintains free Ca^2+^ concentrations of 100–800 μM, which amounts to at least three orders of magnitude higher than in the cytosol (Berridge et al., 2000; Berridge, 2002) (Figure 1A). Therefore, it is remarkable that the ER membrane is not tight to ions; it has indeed a distinct permeability to ions and even small molecules. When the sarcoplasmic/endoplasmic reticulum Ca^2+^ ATP-ases (SERCA), which pump Ca^2+^ into the ER, is blocked, e.g., by thapsigargin, the Ca^2+^ concentration in the ER decreases, unmasking the Ca^2+^ leak/leakage or passive Ca^2+^ efflux from the ER. In the absence of extracellular Ca^2+^, the SERCA inhibition typically leads to a decrease in ER Ca^2+^ with the corresponding transient increase of cytosolic Ca^2+^ (Gamayun et al., 2019). Within several molecular pathways for Ca^2+^ leakage that co-exist in ER membranes, Sec61 translocons are unparalleled because they support both translocation of proteins into the ER and Ca^2+^ leakage from the ER, suggesting a dynamic coupling between ER membrane permeability and protein synthesis (Figure 1B). Therefore, it is not surprising that the Sec61-mediated Ca^2+^ leakage from the ER has been implicated in the etiology of various cancers, neurodegeneration, and infectious diseases (such as Buruli ulcer) as well as inherited diseases, such as immunodeficiency, neutropenia and tubulointerstitial kidney disease (Bolar et al., 2016; Schubert et al., 2018; Van Nieuwenhove et al., 2020; Bhadra et al., 2021; Sicking et al., 2022). Notably, the other ER membrane resident Ca^2+^ leak channels are, in alphabetical order, Bcl-2 (Pinton et al., 2001; Chami et al., 2004), CALHM1 (Gallego-Sandín et al., 2011), Pannexin 1 (Abeele et al., 2006), Presenillins 1 and 2 (Tu et al., 2006), truncated SERCA variants (Chami et al., 2001; Chami et al., 2008) and transient receptor potential superfamily members TRPC1 (Berbey et al., 2009) and TRPP2 (see below). In contrast to the latter proteins, however, the Sec61 translocons are ubiquitous and highly abundant, depending on secretory capacity of the cell, i.e., the extension of the ER (Pick et al., 2021). In HeLa cells, for example, the concentration of heterotrimeric Sec61 complexes is between 139 and 456 nM (judging from the concentration of the subunit with the lowest and highest cellular concentration, respectively, (Lang et al., 2017), and Sec61 channels support about 60% of the Ca^2+^ leakage from the ER (Lang et al., 2011; Gamayun et al., 2019).

Originally, the Ca^2+^ leakage from the ER and specifically, the Sec61-mediated Ca^2+^ leakage from the ER represented a new and unexpected mechanisms of the ER Ca^2+^ homeostasis. It first came up in the early 2,000 years in seminal papers on human cells (Camello et al., 2002; Lomax et al., 2002; Van Coppenolle et al., 2004; Flourakis et al., 2006; Giunti et al., 2007) and, subsequently, was confirmed *in vivo* by a global RNAi screen for genes that are involved in store-operated Ca^2+^ entry (SOCE) in *Drosophila* (Zhang et al., 2006) as well as by biochemical and biophysical approaches (Wirth et al., 2003; Erdmann et al., 2011; Lang et al., 2011; Schäuble et al., 2012). The latter experimental approaches involved single channel recordings from purified and reconstituted Sec61 complexes and live cell calcium imaging in cytosol and ER lumen of human cells in combination with siRNA treatment or plasmid driven mutant variant expression. Several studies also identified various interaction partners of the Sec61 channel that are involved in tight control of the Ca^2+^ leak (Figure 1B), i.e., the ER-lumenal chaperone BiP and its cochaperones ERj3 and ERj6 (Schäuble et al., 2012; Schorr et al., 2015) as well as cytosolic calmodulin (CaM) and the ER membrane protein Sec62 (Erdmann et al., 2011; Linxweiler et al., 2013), thereby preventing excessive Ca^2+^ leakage that may lead to apoptosis (Hara et al., 2013; Feliziani et al., 2020). Furthermore, three inhibitors of the Sec61 channel, Eeyarestatins ES1 and ES24 as well as Mycolactone have been characterized as enhancers of Ca^2+^ leakage (Gamayun et al., 2019; Bhadra et al., 2021). As further readings on the subject of Sec61 inhibitors we recommend recent reports on the cryo-EM structures of the mammalian Sec61 translocon inhibited by various small molecules (Gérard et al., 2020; Itskanov et al., 2022; Rehan et al., 2022).

In the last 5 years, a picture started to evolve in which the Sec61-mediated Ca^2+^ leakage from the ER is not only a major player in various pathophysiological settings but also provides a link between energetic requirements of protein translocation into and folding and assembly within the ER under physiological conditions (Klein et al., 2018; Yong et al., 2019; reviewed by Zimmermann and Lang, 2020). Briefly, human SLC35B1 apparently imports ATP into the ER in exchange for ADP and was named AXER (ATP/ADP exchanger in the ER membrane) (Klein et al., 2018; Schwarzbaum et al., 2022). Furthermore, an ER low energy response (termed lowER) was characterized as a central reulatory circuit for maintaining ATP supply to the ER. This regulatory circuit was proposed to involve BiP dissociation from the Sec61 channel under conditions of a low ATP/ADP ratio in the ER lumen, thus allowing Ca^2+^ leakage from the ER (Klein et al., 2018). Accordingly, Ca^2+^ binds to CaM in the cytosol and activates AMP-activated protein kinase *via* Ca^2+^/CaM dependent kinase 2 and, eventually, 6-phospho-fructo-2-kinase. Activated 6-phospho-fructo-2-kinase stimulates ADP phosphorylation in glycolysis, subsequently allowing ATP import into the ER *via* AXER (in exchange for ADP), which is futher activated by Ca^2+^ efflux from the ER. Normalization of the ER ATP/ADP ratio allows BiP to limit the Ca^2+^ leakage *via* binding to Sec61 channels and thus inactivates the regulatory circuit. However, the details of this signal transduction pathway are still somewhat controversial (Yong et al., 2019; Zimmermann and Lang, 2020).

## Concept and contributions for the Research Topic

Goal of this Research Topic is to present a combination of review articles and state-of-the-art studies that cover aspects of the Ca^2+^ leak from ER in health and diseases. Considering that Sec61 translocons function as ion channels in the ER membrane, it appeared to be interesting to explore the pore structure and eventually the open-closed kinetics of these unusual ion channels. It is remarkable that the Sec61-mediated Ca^2+^ leak from the ER has been implicated in the etiology of diseases such as cancer and inherited as well as infectious diseases. A good proportion of the papers in the Research Topic will therefore focus on ER Ca^2+^ leakage in diseases. Finally, a number of small molecules that inhibit protein translocation have been described and we would like to draw the attention to papers looking for their mode of action with focus on the ER Ca^2+^ leak (Gamayun et al., 2019; Gérard et al., 2020; Bhadra et al., 2021).

In this Research Topic, renowned international experts in the area of cell biology and human medicine report on their mechanistic and medical insights into various aspects of the Ca^2+^ leak/leakage or passive Ca^2+^ efflux from the ER. Schulte and Blum set the stage and provide a comprehensive overview about the Ca^2+^ homeostasis in human neuronal cells, specifically, the surprisingly dynamic Ca^2+^ fluxes between the ER, the cytosol and the extracellular space as well as how the ER Ca^2+^ leak contributes to evolutionary conserved Ca^2+^ phenomena such as SOCE, ER Ca^2+^ induced Ca^2+^ release (CICR) and Ca^2+^ oscillations. Next, Pick et al. present their quantitative data and kinetics of thapsigargin-induced Ca^2+^ efflux from the ER and SOCE as its consequence on the Ca^2+^ dynamics in HEK293 cells. Parys and Van Coppenolle focus our attention on the Sec61 channel as the most abundant and ubiquitous ER Ca^2+^ leak channel and its various roles in health and disease. This brilliant review also introduces the various interaction partners of the Sec61 channel that are involved in tight control of the leak (including Sec62). Dagnino-Acosta and Guerrero-Hernandez add another control mechanism of the Sec61 channel, i.e., phosphorylation, which at least in smooth muscle cells is mediated by protein kinase C (PKC). Coming back to the role of the Sec61 channel and its modulators in disease, the contribution by Zimmermann et al. on tumor diseases is highly recommended to bridge the gap from bench to bedside. This paper is also an excellent introduction for two original articles by Radosa et al. as well as Körner et al. respectively, which deal with Ca^2+^ efflux from the ER as a novel target of anti-metastatic and anti-proliferative therapy in head and neck cancer and the oncogene *SEC62* as a prognostic marker in patients with ovarian malignancies, respectively. Staying with human medicine, the Research Topic is finished off by Liu et al. who discuss the current views on the biophysical and physiological properties of the ER membrane protein PKD2, which is also termed polycystin-2 or TRPP2, and on how PKD2 contributes to ER Ca^2+^ homeostasis in cell physiology and to autosomal polycystic kidney disease in pathophysiology. Notably, PKD2 represents one of the additional channels for Ca^2+^ efflux from the ER, which be highlighted to exist in addition to the Sec61 channel in the Introduction to this editorial.
